# A Primal Analysis System of Brain Neurons Data

**DOI:** 10.1155/2014/348526

**Published:** 2014-07-24

**Authors:** Dong-Mei Pu, Da-Qi Gao, Yu-Bo Yuan

**Affiliations:** School of Information Science and Engineering, East China University of Science and Technology, Shanghai 200237, China

## Abstract

It is a very challenging work to classify the 86 billions of neurons in the human brain. The most important step is to get the features of these neurons. In this paper, we present a primal system to analyze and extract features from brain neurons. First, we make analysis on the original data of neurons in which one neuron contains six parameters: room type, *X*, *Y*, *Z* coordinate range, total number of leaf nodes, and fuzzy volume of neurons. Then, we extract three important geometry features including rooms type, number of leaf nodes, and fuzzy volume. As application, we employ the feature database to fit the basic procedure of neuron growth. The result shows that the proposed system is effective.

## 1. Motivation and Contribution

In February 18, 2013, the Obama administration was planning a decade-long scientific effort to examine the workings of the human brain and build a comprehensive map of its activity (The New York Times reported).

*“Every dollar we invested to map the human genome returned 140 dollars to our economy every dollar,” Obama said. “Today our scientists are mapping the human brain to unlock the answers to Alzheimers. They're developing drugs to regenerate damaged organs, devising new materials to make batteries 10 times more powerful. Now is not the time to gut these job-creating investments in science and innovation.”*

It is well known that brain structure and function are the most complex organization, which contains hundreds of billions of nerve cells (neurons). It is a very challenging work to classify the 86 billions neurons in the human brain. Until now, the human race only discovered the basic information about two thousands of neurons. In 1997, human brain project (HBP) was officially launched in the United States, more than 20 countries took part in the project, such as the United States, Britain, Germany, France, Sweden, Norway, Switzerland, Australia, Japan, and so on. The primal goal of HBP is to study neural information to the database given all over the world. The aim is to establish common standards, multidisciplinary integration of massive data analysis, and accelerate understanding of the human brain. As the basic unit of brain, neurons are very important to the biological behavior of people. Neurons in human brain structure and function play an important role. With the development of biological technology and computer science, people can obtain some detailed data of human brain neurons. But it is a very difficult thing to identify the class of neurons [1–8]. It is the motivation of this work.

In this paper, as the basic step, we present a primal system to analyze the neurons and extract features from the known brain neurons. In Section 2, analysis on six parameters including room type, *X*, *Y*, *Z* coordinate range, total number of leaf nodes, and fuzzy volume of neurons was made. In Section 3, we extract three important geometry features of the known neurons including rooms type, number of leaf nodes, and fuzzy volume. In Section 4, we employ the feature database to fit the basic procedure of neuron growth. Finally, we give a conclusion with this work in the last section.

## 2. Introduction of Neurons Databases and Basic Hypothesis

### 2.1. Introduction of Neurons Databases

In this paper, the data sets are downloaded from the following webs:

1. http://neuromorpho.org/neuroMorpho/LS_availability.jsp
2. http://neuromorpho.org/neuroMorpho/index.jsp.

**Figure 1:**
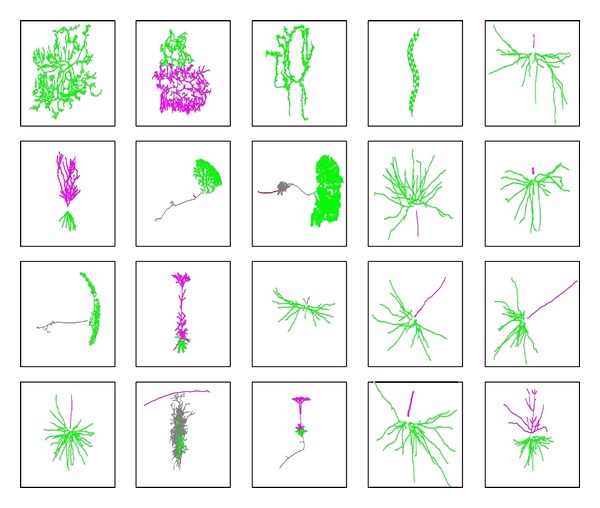


Some figures of interesting neurons are shown in Figure 1.

If the readers or researchers have some difficulty with the data sets, then data sets can be provided to readers by E-mails or downloaded from ftp of our team lab.

*(1) Data Set A.* The data set contains five groups of neuron data: cat motoneuron (5 groups), interneuron (bipolar interneuron (5 groups), multipolar cell (5 groups), and tripolar interneuron (5 groups)), purkinje cell (guinea-pig purkinje cell (3 groups) and mouse purkinje cell (3 groups)), rat pyramidal (7 groups), and sensory neuron (7 groups).

*(2) Data Set B.* This is a prediction set; there are twenty groups of neuron data. And all neurons in data set B are unknown, which are waiting for classification by mathematical model.

*(3) Data Set C.* The data set C is a standard set which contains seven groups of data and every group is a known type of neuron. (Seven neurons are listed in Table 3.)

### 2.2. Basic Hypothesis

We give some basic hypothesis on data.

*(1) Unified Coordinate.* All the data coordinates are given in the same coordinate.

*(2) Similar Capacity.* In data set A, the neurons data contains two kinds: the same neurons of different animals and the neurons in different growth periods of the same animal. However, in data set C, every neuron contains a group data. We determine the classification standard by the data of data set C and the similar capacity data in data set A.

*(3) Without Structure Change.* The influence of the genetic variation of structural in neurons was not considered in the study of the growth process of neurons.

*(4) Same Rooms.* For the same neurons, rooms arrangement is roughly the same; there is no obvious difference.

## 3. Setting Up the System and Solving System

### 3.1. Neurons Data Analysis

For every neuron, it contains large amount of data; in order to get mathematical classification system, we must preprocess the primal data. Firstly, an analysis is made on standard data of rooms; every group data is seven-dimensional. (In Table 1, 12 neurons are listed.) There are

1. label of rooms;
2. type of rooms;(in which 0 denotes pending, 1 denotes soma, 2 denotes axon, 3 denotes dendron, 4 denotes apical dendrite, and so on) (see Table 2);
3. the *X* coordinate of a room;
4. the *Y* coordinate of a room;
5. the *Z* coordinate of a room;
6. radius of a room;
7. label of the mother room (root room) connected with the room.

*X*, *Y*, *Z* and the radius of a room control the size and the position in a whole neuron. The connected sequence between a room and its root room shows the arrangement of rooms in a neuron and affects the sparse density of the arrangement of rooms. The following data is from one part of cat motoneuron.

**Figure 2:**
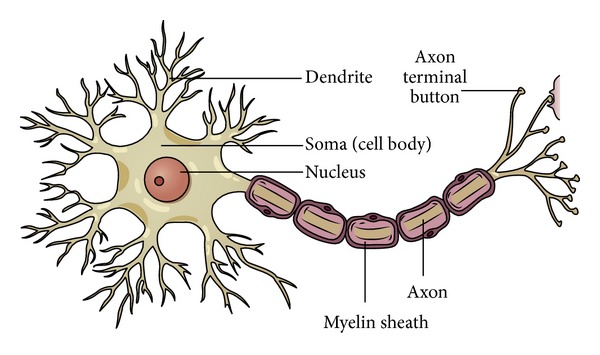
Structure illustration of neuron.

For more knowledge about structure of neuron, we can learn from Figure 2.

Different types of rooms will make up different neurons correspondingly. The biggest value of root room is the most important impactor with the space structures of a neuron. After investigating the connections between rooms and their root rooms, we can determine the number of terminal nodes (also called leaf nodes) in each neuron.

#### 3.1.1. Room Recognition System

First of all, we select the data of data sets A and C, at the same time, different types of rooms with different colors. Consulting standard data, the seventh column, based on the connected sequence of rooms, restores the frame of neurons.

In fact, a neuron contains different types of rooms, but the shape is also different. In another word, the type of room can be used as a parameter of neurons recognition. Let *A* = {0,1, 2,3, 4} be a set of all types of rooms.

There is only one type of room (2) for sensory neurons. Pyramidal neuron has four types of rooms (1, 2, 3, 4). Bipolar interneuron has three types of rooms (1, 2, 3). For motor neuron, purkinje neuron, tripolar interneuron, and multipolar neuron, they have the same type of rooms. They have two types of rooms (1, 3).

According to the above statistical results, we can make the first classification of neurons.

Based on the number of rooms types, we set up the following room recognition system. We let *n* denote the number of types and let *f*(*n*) denote the value used to classify the neurons. The recognition function is given as follows:

(1) $$\begin{array}{c} f\left(n\right)=\{\begin{array}{cc} 7 & n=1;\\ 0 & n=2;\\ 4 & n=3;\\ 3 & n=4;\\ \infty & \text{otherwise}, \end{array} \end{array}$$

where *f*(*n*) = 0 means that the classification is unknown and *f*(*n*) = *∞* means that the primal classification system cannot give any decision.

We process data through statistical methods, which can tell whether a neuron is one among the third type, the fourth type, or the seventh, where the seventh neuron, sensor neuron, is the most easily identified by the room recognition system. It is difficult to judge the rest types only by the room recognition system. Because their rooms types are very similar, in order to distinguish, we need further modeling.

#### 3.1.2. Coordinate Range System

We have already supposed that data which have been used are in the same coordinate system. For investigating neurons shape, we build coordinates range system as follows:

(2) Δx=xmax⁡−xmin⁡;Δy=ymax⁡−ymin⁡;Δz=zmax⁡−zmin⁡.

Coordinate range system can be used to judge neuron shape and coordinate ranges are reliable parameters of space character.

#### 3.1.3. Leaf Nodes System

*Extracting the Feature of Leaf Nodes.* The seventh column in the data set A, data set B, and data set C is the labeled root room. Rooms are housed with primary atrioventricular connection. According to this law, we can get a neuron connected to the atrioventricular arrangement. A neuron in a limited space for all atrioventricular distribution is in three-dimensional space. By the seventh column, we can find all of the leaf nodes (atrioventricular) of neurons and calculate the total leaf nodes [9].

*Algorithm I.* Calculate the total number of leaf nodes.

*Step 1.* Extract the last column of the neurons room data of the data sets A, B, and C.

*Step 2.* Count room serial number. Write down the serial number of room which appears twice or more. Afterwards calculate the total number of rooms in which serial number appeared twice or more by using the symbol *m* to denote them and record the sum of number of repetitions which denote *n*.

*Step 3.* Calculate the total number of leaf nodes *N*, *N* = *n* − *m* + 1.

In the following discussion, unless specified remark, the total number of leaf nodes is simplified as “No.”

#### 3.1.4. Fuzzy Volume System

In this subsection, we calculate a geometry character of neurons called* fuzzy volume*. It is known that difference in shape and size will lead to different neurons. In [10], according to 20 *μ*m thick frozen section on *L*6 back-root DRG of the adult cat, the researchers measured soma diameter to explore the relationship between the size of soma and type of neurons. Eventually, they marked off three types (large, medium, and small). Inspired by this method, we suppose that every room has a specific shape. When a neuron is cut into different rooms, every room is treated as a sphere. In this case, room coordinates are the center section (if not, it can reach the requirements through transformation). After getting radius of all rooms, we can estimate the volume of every room. By the sum of all the volumes of rooms, we can obtain the approximate volume. In practice, it is not the true volume of a neuron. There are some errors. Mathematically, the volume can still be used as an important parameter for classification. The fuzzy volume can be calculated by the following formula:

(3) $$\begin{array}{c} V=\overset{n}{\underset{i=1}{\sum }}\frac{4}{3}\pi r_{i}^{3}, \end{array}$$

where *n* is the total number of rooms and *r*_*i*_ is the radius of the *i*th room.

When we consider the location of rooms in a neuron, there is no existing law that can be used. In fuzzy volume computational system, we suppose that the locations of rooms are similar. By the system, the size can be recognized roughly; in another word, large size or small neurons can be classified easily. We can obtain the fuzzy volume of seven kinds of neurons in data sets A and C (see Table 4).

**Remark 1 .**

But there are different neuron sizes with different ages, especially interneurons; the growth is more complex. So, in this work, for the same kind of neuron, we choose the capacity of samples as research objects. If neurons have the same capacity, we can say that the neurons are at the same age.

### 3.2. The Geometrical Characteristics of Neurons

Based on the above analysis, we consider several geometrical characteristics of neurons. Due to the complexity and variability of neurons, we need to establish a more complete features system to make classification and identification of the neurons. The geometrical characteristics are

1. the type of rooms;
2. *X* coordinate range;
3. *Y* coordinate range;
4. *Z* coordinate range;
5. the total number of leaf nodes;
6. the fuzzy volume of a neuron.

Among them, fuzzy volume is a reliable indicator when considering the growth process. Therefore, till now we set up a complete system for the classification of neurons. We get the following standard data in data set C.

## 4. Some Growth Prediction of Neurons

### 4.1. The Analysis of Neuron Growth

Axon and dendron are main elements of a neuron, which must change greatly along with the growth of a neuron and make a big impact on the shape of a neuron. Under the same criteria for dividing rooms of a neuron, intuitively thinking, when a neuron grew up, the number of rooms will increase in general. Here we do not consider the attenuation of neurons of the adult animal. For the growth of neurons, we choose the data in the data set A to study. The reason is that every kind of neuron in data set A contains several different size samples of rooms data, which can present the change. For the same neurons, rooms sequence does not change daramatically, so researching the fuzzy volume of neurons is meaningful. For our classification feature system, the number of rooms (number 1) and the total number of leaf nodes (number 2) of a neuron can be used to predict the growth of neurons. The more the samples of the same neuron are, the more our prediction will get an accurate result, so we chose the multipolar interneurons data in data set A as the research object. It has nine groups of data; it may reflect the growth trend more accurately.

### 4.2. Data Fitting

According to the data of Tables 5 and 6, we select three arrays as follows: number of rooms, fuzzy volume; number of leaf nodes, fuzzy volume; number of leaf nodes, number of rooms. According to the corresponding data using least-square fitting predicts the change trend of morphological characteristics of multipolar interneuron. The fitting functions, respectively, are as follows [11–14]:

(4) y=0.9x2−59x+1255.1;y=−5x4+139x3−1985x2+14340x−36074;y=−0.3x4+9.1x3−110.6x2+631.4x−1268.4.

### 4.3. Analysis on the Result of Data Fitting

**Figure 3:**
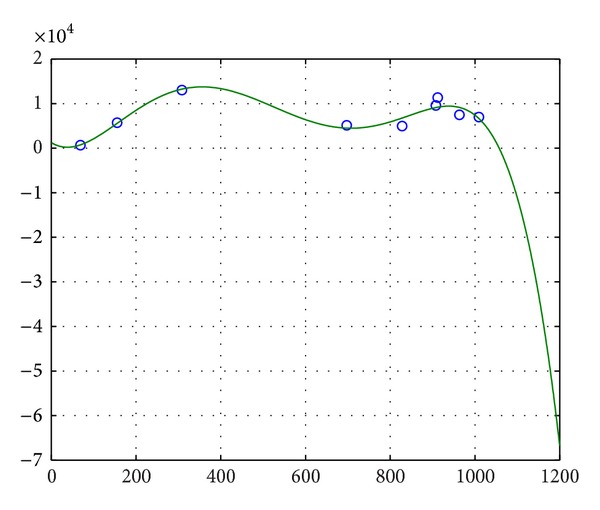
Number of rooms and fuzzy volume.

**Figure 4:**
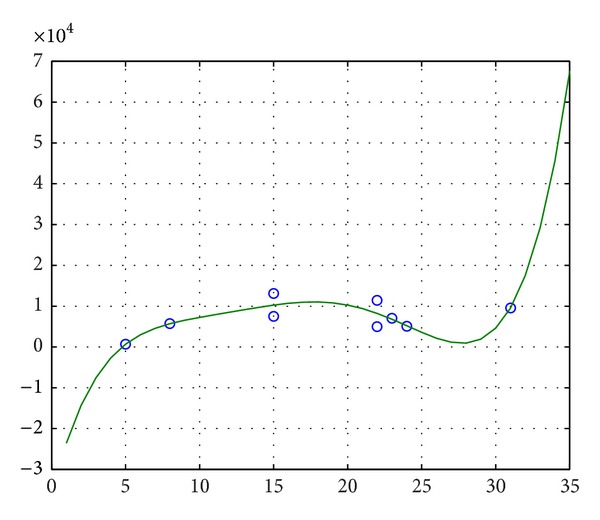
Number of leaf nodes and fuzzy volume.

**Figure 5:**
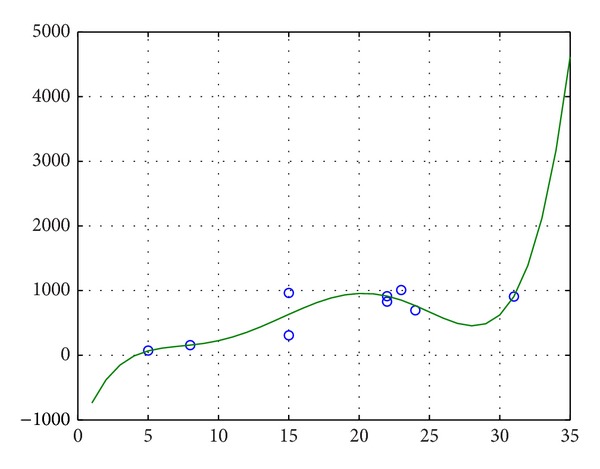
Number of leaf nodes and number of rooms.

1. The fuzzy volume of every neuron is nonnegative. Every neuron has life cycle; therefore, the total number of rooms may not always increase in the whole life. From Figure 3, the fuzzy volume curve changes in a certain interval. We can predict that the fuzzy volume has growing trend from childhood to adulthood for a neuron. After a certain degree, the trend will no longer exist.
2. As life cycles of neurons, the number of leaf nodes has upper bound. From Figure 4, along with increasing of the number of leaf nodes, the fuzzy volume generally increases. We know this kind increase will stop at a certain time.
3. From Figure 5, along with increasing of the number of leaf nodes, the number of rooms of a neuron generally increases. Similarly, this trend will stop after a certain time.

Because the growth process of a neuron is a life cycle from childhood to adulthood, the process of changing cannot be infinite. Because the samples for data fitting are limited, the trend only partly presents the change of the neuron. In order to improve the credibility, we need more samples to be tested for data fitting.

## 5. Conclusion

In this paper, a data analysis and feature extraction system for brain neurons are proposed. The first step is to analyze the original data of neurons. The concept of fuzzy volume of neurons is presented. Six parameters are selected as the important indexes. They are room type, *X*, *Y*, *Z* coordinate range, total number leaf nodes, and fuzzy volume of neurons. After preprocessing, three important geometry features including rooms type, number of leaf nodes, and fuzzy volume are extracted as the basic elements of the brain neuron database. The feature database is very useful for brain neurons classification.

## Figures and Tables

**Table 1:** 

(1)	(2)	(3)	(4)	(5)	(6)	(7)
1	1	0	0	0	51.7	−1
2	3	2	−85	−123.9	7.345	1
3	3	−2	−95	−131.20	4.775	2
4	3	2	−120	−137.1	3.99	3
5	3	−13	−146	−113.80	3.33	4
6	3	13	−131	−200.8	2.005	5
7	3	22	−149	−184	1.910	6
8	3	18	−187	−257	1.81	7
9	3	31	−211	−310	1.805	8
10	3	32	−258	−389	0.61	9
11	3	−1	−266	−463	0.53	10
12	3	−9	−279	−448.6	0.2050	11

**Table 2:** The type of neuron room.

Type of room	Room Name
0	pending
1	soma
2	axon
3	dendron
4	apical dendrite

**Table 3:** Standard data in data set C.

Label	*f*	*X* range	*Y* range
C1	0	3257	1153
C2	0	133.82	124.65
C3	3	560	791
C4	4	281.23	1102.93
C5	0	189.02	366.73
C6	0	329.03	378.45
C7	7	111.31	139.26

**Table 4:** Fuzzy volume of standard data in data set C.

Label	*Z* range	No.	Fuzzy volume
C1	2377	166	500530
C2	8.5	344	2071.6
C3	400	93	5617.5
C4	139.15	66	944.846
C5	21.3	13	5475.4
C6	33.18	25	12291
C7	70.43	12	1139.2

**Table 5:** Standard data in data set A.

Label	No. 1	Volume	*X*
A61	697	5105.3	240.04
A62	69	678.6647	37.8
A63	828	4940.8	264.97
A64	1009	7002.2	263.12
A65	155	5700.7	84.48
A66	912	11380	510.69
A67	308	13060	123.62
A68	908	9546.1	369.04
A69	963	7464.1	329.83

**Table 6:** Standard data in data set A.

Label	*Y*	*Z*	No. 2	Type
A61	214.48	37.97	24	6
A62	37.48	0	5	6
A63	275.05	72.42	22	6
A64	338.63	47.54	23	6
A65	120.79	41.45	8	6
A66	246.31	38.17	22	6
A67	245.25	29.57	15	6
A68	270.4	71.35	31	6
A69	307.17	36.42	15	6
